# Development and Internal Validation of Interpretable Machine Learning Models for Identifying Burnout Syndrome Among Intensive Care Unit Nurses

**DOI:** 10.1155/jonm/6835251

**Published:** 2026-07-19

**Authors:** Wei Hu, Yunfan Ji, Fengzhi Chai, Di Xu, Yuhong Wang, Caiyue Xu, Xia Li

**Affiliations:** ^1^ Department of Nursing, First Affiliated Hospital of Jinzhou Medical University, Jinzhou, Liaoning, China, jzmu.edu.cn; ^2^ School of Nursing, Jinzhou Medical University, Jinzhou, Liaoning, China, jzmu.edu.cn

**Keywords:** burnout, intensive care unit, machine learning, nurses, psychological resilience, random forest

## Abstract

**Background:**

Burnout among intensive care unit (ICU) nurses threatens patient safety and healthcare quality. We aimed to develop and internally validate a machine learning model to identify current burnout and its key correlates in this population.

**Methods:**

We surveyed 318 ICU nurses across four tertiary hospitals in three provinces of China (October 2024–November 2024), measuring 34 potential predictor variables. Data were partitioned into training (70%) and testing (30%) sets with downsampling addressing class imbalance. LASSO regression identified 12 significant predictors, which were evaluated using 10 machine learning algorithms. The final model was assessed using AUC, calibration, and SHAP analysis.

**Results:**

The random forest algorithm showed optimal performance, with the final nine‐predictor model achieving an AUC of 0.983, with good calibration (Brier score 0.054). SHAP analysis revealed psychological resilience (0.197) and job satisfaction (0.152) as primary protective factors, while nursing stress (0.059), night shift frequency (0.016), and poor sleep quality (0.015) emerged as key risk factors. Marital status, commuting mode, children, and residential area contributed additionally to predictions.

**Conclusion:**

The internally validated classification model developed in this study suggests that psychological resilience, job satisfaction, and nursing stress may play important roles in ICU nurse burnout. These findings can help nurse managers target organizational interventions—such as adequate staffing, recovery‐protective scheduling, and support for resilience and job satisfaction—to prevent burnout, rather than placing responsibility on individual nurses. Further validation of this tool in diverse healthcare settings would be beneficial.

## 1. Introduction

Burnout syndrome, characterized by emotional exhaustion (EE), depersonalization (DP), and reduced personal accomplishment (PA), represents a significant occupational hazard among healthcare professionals worldwide [1]. Initially conceptualized by Maslach et al. [2], this syndrome emerges as a psychological response to chronic workplace stressors. More recent work has characterized the biological consequences of chronic occupational stress through the allostatic‐load framework, which describes the cumulative physiological cost of repeated stress activation and incomplete recovery [3]. While we use the Maslach Burnout Inventory (MBI) to remain comparable with the extensive burnout literature, we recognize this broader neurophysiological perspective on prolonged work stress. Among healthcare providers, nurses working in intensive care units (ICUs) face particularly high risks of developing burnout due to their exposure to high‐stress environments, life‐and‐death situations, ethical dilemmas, and complex patient care demands [4, 5]. The prevalence of burnout among ICU nurses ranges from 25% to 80% globally, with alarming rates reported across diverse healthcare systems [6]. A systematic review by Chuang et al. revealed that ICU nurses experience significantly higher burnout levels compared to nurses in other specialties [7], with rates often exceeding 50% in Asian countries [8]. This high prevalence is particularly concerning given China’s healthcare system challenges, including nurse shortages and increasing patient demands in critical care settings [9, 10]. The consequences of burnout extend beyond individual nurses’ well‐being to significantly impact healthcare delivery and patient outcomes. Studies have established robust associations between nurse burnout and increased medical errors, healthcare‐associated infections, patient mortality, and reduced patient satisfaction [11, 12]. Li et al. demonstrated that ICU nurse burnout correlates with decreased patient safety indicators and compromised quality of care [13]. Furthermore, burnout contributes substantially to nurse turnover intentions, absenteeism, and workforce instability, exacerbating existing healthcare workforce challenges [14, 15]. Despite recognition of the multifactorial etiology of burnout, traditional research approaches have often examined risk factors in isolation, limiting the development of comprehensive predictive models [16]. Current literature identifies various potential contributors to ICU nurse burnout, including work environment factors (workload, staffing adequacy, and interprofessional relations) [17, 18], personal characteristics (psychological resilience and coping mechanisms) [19, 20], and organizational variables (leadership, professional autonomy, and recognition) [21, 22]. However, the complex interplay between these factors remains inadequately understood, hampering the development of effective preventive interventions. Machine learning (ML) offers promising approaches for addressing these limitations through its capacity to identify complex patterns within multidimensional data [23]. ML algorithms have demonstrated superior predictive performance compared to conventional statistical methods across various healthcare domains [24, 25]. Recent applications in mental health risk prediction have yielded encouraging results, with studies showing high accuracy in identifying individuals at risk for psychological distress [26]. The few existing ML models for healthcare worker burnout have typically relied on limited predictor sets or single‐center data, restricting their generalizability and clinical utility. Additionally, many models lack interpretability—a critical limitation for implementation in real‐world healthcare settings where understanding contributing factors is essential for intervention design [27]. Mehta et al. emphasized that clinical implementation of ML models requires both predictive accuracy and interpretability to facilitate targeted interventions [28]. The significance of this work lies in its potential to enable identification of nurses currently experiencing burnout and inform targeted preventive interventions. By identifying key modifiable risk factors and their relative contributions to burnout probability, this study provides actionable insights for healthcare administrators and policymakers to allocate resources effectively and design evidence‐based prevention strategies [29]. Furthermore, the methodological approach demonstrates the value of ML in addressing complex healthcare workforce challenges.

## 2. Methods

### 2.1. Study Population

This cross‐sectional study was conducted between October 2024 and November 2024 across four tertiary (Grade A) hospitals in four cities across three provinces of China: Shangrao and Nanchang (Jiangxi Province), Jinzhou (Liaoning Province), and Fuzhou (Fujian Province). The participating hospitals were enrolled by convenience sampling, and all eligible ICU nurses at each site were invited to participate. The participating units comprised a mix of ICU types, including general, neurological, respiratory, emergency, and cardiothoracic ICUs; the participating ICUs therefore differed by geographic region and by ICU type, although all were located within Grade A tertiary hospitals. The study protocol was approved by the Institutional Review Board of Jinzhou Medical University (approval number: JZMULL2024136), and all participants provided informed consent prior to enrollment. Participants were eligible if they were registered nurses currently working in ICUs (medical, surgical, comprehensive, or specialized ICU) with a minimum of three months of continuous ICU work experience, employed full‐time (≥ 40 h per week) with direct patient care responsibilities, able to understand and complete the questionnaire independently, and willing to provide informed consent. We excluded nurses in management positions without regular direct patient care responsibilities, temporary or floating staff without permanent ICU assignment, nurses on extended leave, student nurses, individuals with prior diagnosis of severe psychiatric disorders, those with incomplete questionnaire responses, and nurses who had participated in another burnout intervention study within the previous 6 months.

A total of 350 questionnaires were distributed to eligible ICU nurses across the participating hospitals, with 318 valid and complete questionnaires returned (response rate: 90.9%). Following established ML principles, we employed least absolute shrinkage and selection operator (LASSO) regression for feature selection, reducing the initial 34 potential predictors to ≤ 10 key variables in the final model [30]. This approach yielded a sample‐to‐feature ratio exceeding 31:1, substantially surpassing the minimum recommended threshold of 10:1 for predictive modeling [31, 32]. Sample size calculation indicated that with 318 participants, our study achieved statistical power > 0.99 for detecting medium effect sizes (*f* ^2^ = 0.15) at *α* = 0.05 in the predictive modeling analyses. To maximize model robustness, we implemented 10‐fold cross‐validation during model training and evaluation, complemented by SHapley Additive exPlanations (SHAP) analysis to enhance interpretability while maintaining predictive performance [33].

### 2.2. Measurement Instruments

#### 2.2.1. Burnout Assessment

Burnout was assessed using the Chinese version of the MBI [34, 35], which evaluates three dimensions: EE, DP, and reduced PA. The Chinese version demonstrated high internal consistency (Cronbach’s *α* = 0.93), with acceptable reliability in the current study (*α* = 0.86). For the purpose of predictive modeling, burnout was operationalized as a binary outcome in line with Maslach’s multidimensional conceptualization of burnout as a syndrome rather than elevation in any single dimension [36] Using the standard MBI thresholds for healthcare professionals identified in the systematic review by the authors in [37], a nurse was classified as experiencing burnout only when high‐risk thresholds were simultaneously met across all three dimensions: EE ≥ 27, DP ≥ 10, and reduced PA ≤ 33. This stringent joint criterion was adopted to ensure that the modeled outcome reflects the full burnout syndrome rather than isolated symptom elevation, thereby preserving fidelity to the multidimensional construct. Because the study was cross‐sectional, all predictors and the burnout outcome were measured concurrently; the resulting model therefore classifies current (prevalent) burnout status rather than predicting the future development of burnout.

#### 2.2.2. Moral Distress Measurement

The Chinese version of the Moral Distress‐Appraisal Scale (MD‐APPS) [38, 39] was used to assess participants’ moral distress levels. The translated scale demonstrated acceptable reliability (Cronbach’s *α* = 0.74), which was maintained in the current study (*α* = 0.79).

### 2.3. Job Satisfaction

Nurse satisfaction was evaluated using the Chinese version of the McCloskey/Mueller Satisfaction Scale (MMSS) [40, 41]. This instrument has shown high reliability of the Chinese version (Cronbach’s *α* = 0.95), with good reliability maintained in the present study (*α* = 0.86).

### 2.4. Work‐Related Stress

The Chinese version of the Nursing Stress Scale [42, 43] was employed to measure perceived work stress among participants. The Chinese version demonstrated high reliability (Cronbach’s *α* = 0.93), which was maintained in the current study (*α* = 0.91).

### 2.5. Psychological Resilience

Psychological resilience was assessed using the Chinese version of the Connor–Davidson Resilience Scale (CD‐RISC) [44, 45]. The Chinese version demonstrated high reliability (Cronbach’s *α* = 0.91), which was maintained in the current investigation (*α* = 0.90).

### 2.6. Sleep Quality

Sleep quality was evaluated using the Chinese version of the Pittsburgh Sleep Quality Index (PSQI) [46, 47]. The Chinese version demonstrated good reliability (Cronbach’s *α* = 0.84), with acceptable reliability in the current study (*α* = 0.70).

### 2.7. Data Collection and Processing

Demographic characteristics, work‐related information, and psychological scale measurements were collected via questionnaires (see Table 1). To ensure methodological rigor and identify key predictors of burnout among ICU nurses, we implemented a strict data isolation protocol combined with class imbalance correction and regularized regression. Using the createDataPartition function from the caret package in R, we first divided the original dataset into completely nonoverlapping training (70%, *n* = 223) and testing (30%, *n* = 95) sets through stratified random sampling. This critical data isolation step ensured that the test set remained completely separate from all feature selection processes, preventing data leakage and providing an internal assessment of model performance. Because questionnaires with incomplete responses had already been excluded during data screening (see eligibility criteria), the 318 retained questionnaires contained no missing values. The 70%/30% partition therefore yielded complete training (*n* = 223) and test (*n* = 95) sets, and no observations were removed by complete‐case analysis.

**TABLE 1:** Baseline characteristics of ICU nurses by burnout status.

Characteristics	Subgroups	Nonburnout (*n* = 114)	Burnout (*n* = 204)	*p* value
Department (%)				< 0.001
	General ICU	87 (76.3)	94 (46.1)	
	Neurological ICU	20 (17.5)	58 (28.4)	
	Respiratory ICU	2 (1.8)	23 (11.3)	
	Emergency ICU	5 (4.4)	20 (9.8)	
	Cardiac‐thoracic ICU	0 (0.0)	9 (4.4)	
Age (years) (median [IQR])		29.00 [25.00, 32.00]	31.00[28.00, 34.00]	< 0.001
Gender = female (%)		85 (74.6)	180 (88.2)	0.003
Marital status (%)				< 0.001
	Single	73 (64.0)	76 (37.3)	
	Married	40 (35.1)	125 (61.3)	
	Cohabiting	0 (0.0)	2 (1.0)	
	Divorced	1 (0.9)	1 (0.5)	
Education level (%)				0.980
	Technical secondary	2 (1.8)	4 (2.0)	
	Junior college	37 (32.5)	65 (31.9)	
	Bachelor’s degree	73 (64.0)	130 (63.7)	
	Master’s degree	130 (63.7)	130 (63.7)	
Children = yes (%)		34 (29.8)	112 (54.9)	< 0.001
Family structure (%)				< 0.001
	Living alone	40 (35.1)	29 (14.2)	
	Living with partner	38 (33.3)	109 (53.4)	
	Living with parents	33 (28.9)	61 (29.9)	
	Living with children	3 (2.6)	5 (2.5)	
Primary income earner (%)				0.066
	Yes	22 (19.3)	37 (18.1)	
	No	42 (36.8)	52 (25.5)	
	Shared responsibility	50 (43.9)	115 (56.4)	
Monthly income (%)				0.001
	< 3000 CNY	13 (11.4)	6 (2.9)	
	3001–5000 CNY	44 (38.6)	49 (24.0)	
	5001–8000 CNY	39 (34.2)	116 (56.9)	
	8001–12000 CNY	18 (15.8)	33 (16.2)	
Registered residence (%)				0.085
	Local city	65 (57.0)	138 (67.6)	
	Other city in province	24 (21.1)	27 (13.2)	
	City in other province	6 (5.3)	4 (2.0)	
	Rural area	19 (16.7)	35 (17.2)	
Living area (%)				0.833
	Near hospital (< 30‐min walk)	51 (44.7)	89 (43.6)	
	Urban area (< 1‐h commute)	52 (45.6)	100 (49.0)	
	Urban area (1–2‐h commute)	3 (2.6)	3 (1.5)	
	Urban area (> 2‐h commute)	8 (7.0)	12 (5.9)	
Commuting mode (%)				0.009
	Walking	21 (18.4)	14 (6.9)	
	Bicycle/E‐bike	61 (53.5)	112 (54.9)	
	Public transport	11 (9.6)	22 (10.8)	
	Private car	21 (18.4)	56 (27.5)	
ICU experience (years) (median [IQR])		3.00 [1.50, 5.38]	6.00 [4.82, 8.10]	< 0.001
Professional title (%)				< 0.001
	Nurse	35 (30.7)	22 (10.8)	
	Primary nurse	55 (48.2)	121 (59.3)	
	Supervisor nurse	22 (19.3)	56 (27.5)	
	Associate chief nurse	2 (1.8)	5 (2.5)	
Experience in other departments = yes (%)		61 (53.5)	99 (48.5)	0.463
Night shifts per month (median [IQR])		8.00 [5.25, 9.00]	9.00 [6.00, 10.00]	0.013
Teaching experience = no (%)		41 (36.0)	114 (55.9)	0.001
Ward bed count (median [IQR])		14.00 [10.00, 20.00]	14.00 [10.00, 23.00]	0.614
Patients per shift (median [IQR])		2.00 [2.00, 3.00]	3.00 [2.00, 3.00]	< 0.001
Weekly working hours (median [IQR])		40.00 [40.00, 46.00]	40.00 [40.00, 45.00]	0.770
Rest time adequacy (%)				< 0.001
	Very adequate	22 (19.3)	7 (3.4)	
	Adequate	31 (27.2)	59 (28.9)	
	Neutral	34 (29.8)	65 (31.9)	
	Inadequate	23 (20.2)	58 (28.4)	
	Very inadequate	4 (3.5)	15 (7.4)	
Shift pattern (%)				0.049
	8‐h shift	72 (63.2)	158 (77.5)	
	12‐h shift	35 (30.7)	36 (17.6)	
	16‐h shift	4 (3.5)	6 (2.9)	
	24‐h shift	3 (2.6)	4 (2.0)	
Average consecutive working days (median [IQR])		4.00 [2.00, 5.00]	5.00 [4.00, 5.00]	< 0.001
Break time per shift (%)				0.053
	0–15 min	14 (12.3)	9 (4.4)	
	16–30 min	51 (44.7)	94 (46.1)	
	31–45 min	19 (16.7)	53 (26.0)	
	46–60 min	23 (20.2)	37 (18.1)	
	> 60 min	7 (6.1)	11 (5.4)	
Annual leave days (%)				0.128
	0–5 days	79 (69.3)	112 (54.9)	
	6–10 days	27 (23.7)	74 (36.3)	
	11–15 days	6 (5.3)	11 (5.4)	
	16–20 days	2 (1.8)	6 (2.9)	
	> 21 days	0 (0.0)	1 (0.5)	
Monthly rest days (%)				0.006
	0–4 days	10 (8.8)	15 (7.4)	
	5–8 days	70 (61.4)	159 (77.9)	
	9–12 days	21 (18.4)	22 (10.8)	
	13–16 days	13 (11.4)	8 (3.9)	
Music hobby = yes (%)		65 (57.0)	91 (44.6)	0.045
Sports hobby = yes (%))		39 (34.2)	78 (37.7)	0.361
Moral Distress Scale total score (median [IQR])		27.50 [23.00, 34.00]	27.00 [25.00, 31.00]	0.778
Nurse Stress Scale total score (median [IQR])		32.50 [25.25, 43.75]	51.50 [46.00, 57.25]	< 0.001
Connor–Davidson Resilience Scale total score (median [IQR])		80.00 [71.25, 90.75]	50.00 [42.00, 54.00]	< 0.001
McCloskey/Mueller Satisfaction Scale total score (median [IQR])		120.50 [107.00, 142.00]	90.00 [85.00, 95.00]	< 0.001
Perceived Social Support Scale total score (median [IQR])		72.00 [63.00, 77.75]	57.00 [48.00, 67.00]	< 0.001
Pittsburgh Sleep Quality Index total score (median [IQR])		10.00 [6.00, 13.00]	8.00 [6.00, 10.00]	0.004

*Notes:* Burnout was assessed using the Maslach Burnout Inventory (MBI); nonburnout = 0, burnout = 1. Department types: general ICU, neurological ICU, respiratory ICU, emergency ICU, and cardiothoracic ICU. Educational levels ranged from technical secondary to master’s degree. Professional titles included nurse, primary nurse, supervisor nurse, and associate chief nurse. Psychometric scales: Moral Distress Scale (higher scores indicate greater moral distress); Nurse Stress Scale (higher scores indicate higher stress levels); Connor–Davidson Resilience Scale (CD‐RISC; higher scores indicate greater resilience); McCloskey/Mueller Satisfaction Scale (MMSS; higher scores indicate greater job satisfaction); Perceived Social Support Scale (PSSS; higher scores indicate greater perceived support); Pittsburgh Sleep Quality Index (PSQI; higher scores indicate poorer sleep quality). *p* values were calculated using *χ* ^2^ test or Fisher’s exact test for categorical variables and the *t*‐test or Mann–Whitney *U* test for continuous variables. Continuous variables are presented as median (interquartile range) or mean ± standard deviation as appropriate.

Abbreviations: ICU, intensive care unit; CNY, Chinese uuan.

**FIGURE 1:**
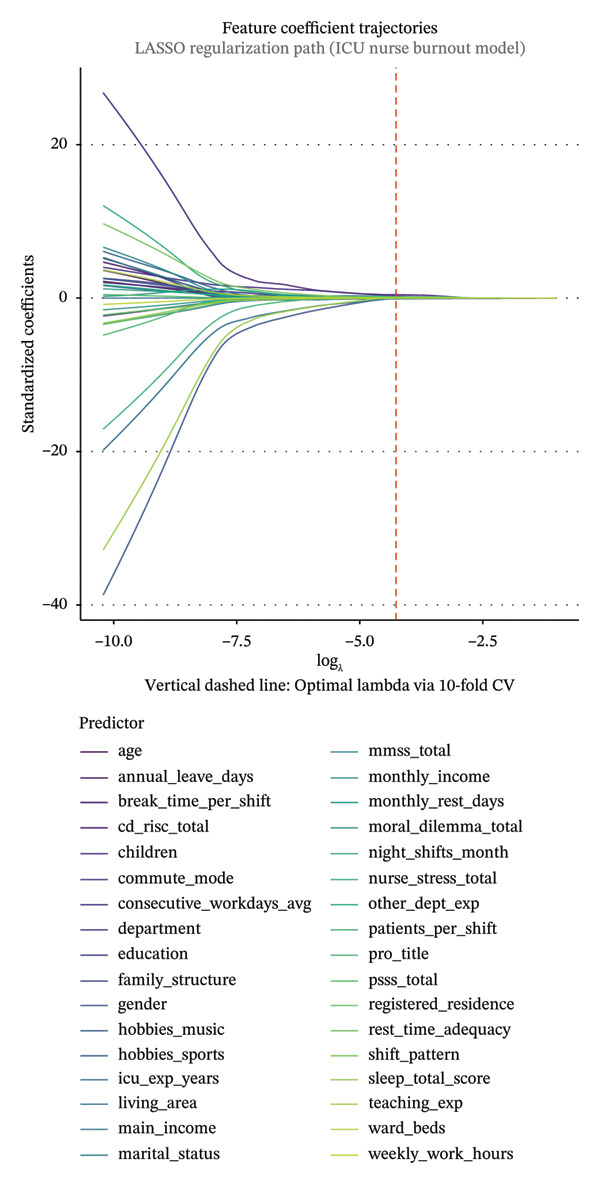
Feature coefficient trajectories in LASSO regularization path for the ICU nurse burnout prediction model. The vertical dashed line indicates optimal lambda value determined by 10‐fold cross‐validation. Variables with nonzero coefficients at this point were selected for the final model.

The training cohort exhibited class imbalance, with burnout cases comprising 61.9% (*n* = 138) and nonburnout cases 38.1% (*n* = 85). To mitigate potential bias toward the majority class, we implemented inverse frequency weighting exclusively within the training set, assigning weights of 1.31 for nonburnout observations and 0.81 for burnout cases. For feature selection and model development, we applied LASSO logistic regression solely on the training data, with an L1 penalty term systematically eliminating irrelevant predictors (see Figure 1). The optimal regularization parameter (*λ* = 0.01469) was determined through 10‐fold cross‐validation on the weighted training data, minimizing binomial deviance while maintaining the integrity of the data isolation protocol. This process identified 12 significant predictors from the original feature set, with positive coefficients indicating factors increasing burnout risk (including marital status, children, monthly income, commuting mode, night shifts, ward bed count, adequacy of rest time, and nurse stress) and negative coefficients indicating protective factors (proximity of residence, psychological resilience, job satisfaction, and sleep quality).

### 2.8. Model Development and Comparison

Following feature selection through LASSO regression that identified 12 significant predictors, we systematically evaluated multiple ML algorithms to develop an optimal prediction model for ICU nurse burnout. Ten distinct classification algorithms were implemented and compared: logistic regression, random forest, extreme gradient boosting (XGBoost), gradient boosting machine (GBM), neural network, adaptive boosting (AdaBoost), decision tree (DT), C5.0, support vector machine (SVM), and naive Bayes. All models were trained exclusively on the training dataset (*n* = 223) using the same 12 predictor variables to ensure fair comparison. To optimize each algorithm’s performance, we employed grid search with 10‐fold cross‐validation to systematically identify optimal hyperparameter configurations. Hyperparameters were tuned for each algorithm according to their specific requirements, with optimization primarily targeted toward maximizing AUC as the main criterion. AUC was chosen as the primary metric due to its threshold‐independent nature, robustness to class imbalance, and direct measurement of discrimination ability. However, final model selection was based on comprehensive evaluation across all performance metrics including sensitivity, specificity, accuracy, F1 score, precision, and negative predictive value (NPV) (Table 2).

**TABLE 2:** Performance metrics (95% CI) of machine learning models for predicting professional burnout risk in ICU nurses.

Model	AUC (95% CI)	Recall (95% CI)	Accuracy (95% CI)	F1 score (95% CI)	Precision (95% CI)	NPV (95% CI)
Logistic	0.961 (0.924–0.998)	0.985 (0.907–0.999)	0.937 (0.862–0.974)	0.956 (0.921–0.985)	0.929 (0.834–0.973)	0.960 (0.777–0.998)
Random forest	0.972 (0.944–1.000)	0.970 (0.885–0.995)	0.916 (0.836–0.960)	0.941 (0.904–0.977)	0.914 (0.816–0.965)	0.920 (0.725–0.986)
XGBoost	0.958 (0.907–1.000)	0.955 (0.864–0.988)	0.895 (0.811–0.946)	0.926 (0.884–0.963)	0.900 (0.799–0.955)	0.880 (0.667–0.968)
GBM	0.955 (0.905–1.000)	0.970 (0.885–0.995)	0.916 (0.836–0.960)	0.941 (0.904–0.977)	0.914 (0.816–0.965)	0.920 (0.725–0.986)
Neural network	0.951 (0.907–0.995)	0.985 (0.907–0.999)	0.937 (0.862–0.974)	0.956 (0.921–0.985)	0.929 (0.834–0.973)	0.960 (0.777–0.998)
AdaBoost	0.970 (0.935–1.000)	0.970 (0.885–0.995)	0.926 (0.849–0.967)	0.948 (0.912–0.978)	0.928 (0.832–0.973)	0.923 (0.734–0.987)
DT	0.929 (0.867–0.992)	0.970 (0.885–0.995)	0.937 (0.862–0.974)	0.955 (0.919–0.985)	0.941 (0.849–0.981)	0.926 (0.742–0.987)
C5.0	0.932 (0.867–0.998)	0.970 (0.885–0.995)	0.905 (0.823–0.953)	0.934 (0.897–0.970)	0.901 (0.802–0.956)	0.917 (0.715–0.985)
SVM	0.920 (0.858–0.983)	0.970 (0.885–0.955)	0.916 (0.836–0.960)	0.941 (0.904–0.977)	0.914 (0.816–0.965)	0.920 (0.725–0.986)
Naive Bayes	0.938 (0.884–0.992)	0.979 (0.921–1.000)	0.926 (0.874–0.979)	0.948 (0.907–0.985)	0.928 (0.857–0.985)	0.923 (0.808–1.000)

Model performance was evaluated using a comprehensive set of metrics to assess different aspects of predictive capability: AUC with 95% confidence intervals (CIs) for discrimination ability, sensitivity/recall (identification of true burnout cases), specificity (correct identification of non‐burnout cases), accuracy (overall correct classification rate), F1 score (harmonic mean of precision and recall), precision (positive predictive value), and NPV. To ensure robust performance estimation and minimize overfitting, all models underwent rigorous 10‐fold cross‐validation during both the hyperparameter optimization and final evaluation phases. Final model assessment was conducted on the completely isolated test dataset (*n* = 95) that remained untouched during all prior model development steps, providing an internal estimate of model performance; external validation in an independent cohort was not performed.

### 2.9. Feature Selection and Model Interpretation

To enhance model transparency and interpretability, we implemented the SHAP methodology [48], a technique designed to rank input feature importance and explain prediction model results while overcoming the “black box” nature of complex ML algorithms. We chose SHAP for feature selection based on its theoretical foundation in cooperative game theory, ability to capture feature interactions in tree‐based models, and provision of both global and local interpretability [49]. Using the 12 features previously identified through LASSO regularization, we calculated mean absolute SHAP values (see Supporting Figure 1) based on our optimal random forest model. These values quantify the average magnitude of each feature’s contribution to predictions across all observations, providing a robust metric for feature importance ranking.

Based on this importance ranking, we conducted a systematic feature reduction analysis to determine the optimal number of features for the random forest model. The feature selection strategy was guided by three key principles: (1) maintaining predictive performance as measured by AUC, (2) achieving model parsimony to enhance clinical interpretability and implementation feasibility, and (3) preventing overfitting through dimensionality reduction [50].

Feature importance was ranked using SHAP values derived from the training data only, and predictors were sequentially eliminated in ascending order of importance to create a series of models with progressively fewer features. The entire reduction procedure was conducted within the training data using 10‐fold cross‐validation, so that the test set played no part in feature selection. At each step, the discrimination of the reduced model was compared with that of the full 12‐feature model using DeLong’s test applied to the pooled out‐of‐fold predictions. The final feature set was selected according to two criteria: (a) statistical noninferiority—no statistically significant decrease in cross‐validated AUC relative to the full 12‐feature model (*p* > 0.05 by DeLong’s test [51]); and (b) parsimony—selection of the smallest feature subset that satisfied Criterion (a), thereby achieving a substantial reduction in model complexity (≥ 25% fewer predictors) without a significant loss of discrimination. This procedure identified the most parsimonious subset whose cross‐validated performance remained statistically equivalent to that of the full model, while further reduction produced a statistically significant decline in discrimination.

The SHAP framework provided both global and local interpretability for our optimal model. Global interpretation generated consistent and accurate attribution values for each feature in the model, revealing the strength and directionality of associations between input features and ICU nurse burnout. This allowed us to quantify the magnitude of influence for each risk factor and protective factor across the entire population. Complementing this, local interpretation enabled individualized predictions by demonstrating how specific input data points contributed to the classification of individual nurses. This dual‐level interpretability framework facilitated both population‐level insights and individual‐level interpretation, enhancing the practical utility of the prediction model in clinical settings.

### 2.10. Statistical Analysis

Continuous variables with non‐normal distributions were presented as median (IQR) and compared using the Mann–Whitney *U* tests or Kruskal–Wallis H tests for multiple categories. Categorical variables were expressed as percentages and analyzed with chi‐square or Fisher’s exact tests. Baseline characteristics were compared between burnout and nonburnout groups, with detailed results presented in Table 1.

Model discrimination was assessed using the area under the receiver operating characteristic curve (AUC), with the optimal classification threshold determined by maximizing the Youden index (sensitivity + specificity − 1). Model calibration was assessed on the test set using a loess‐smoothed calibration curve together with the calibration slope, calibration intercept, and the Brier score. Clinical utility was evaluated through decision curve analysis, quantifying the net benefit of the model across threshold probabilities relative to the “treat all” and “treat none” strategies. SHAP values were calculated using the “shapr” package. For feature reduction, models with different numbers of predictors were compared within the training data using 10‐fold cross‐validation, with DeLong’s test applied to the pooled out‐of‐fold predictions via the “pROC” package. All analyses were performed using R Version 4.4.2, with statistical significance defined as two‐tailed *p* < 0.05.

## 3. Results

### 3.1. Baseline Characteristics of ICU Nurses

Among the 318 ICU nurses included in our study, 204 (63.9%) met the criteria for burnout according to the MBI, while 114 (36.1%) did not exhibit burnout symptoms. As shown in Table 1, significant demographic and professional differences existed between the two groups. Nurses in the burnout group were generally older (median 31.0 vs. 29.0 years, *p* < 0.001) and had longer ICU experience (median 6.0 vs. 3.0 years, *p* < 0.001). The burnout group included a significantly higher proportion of female nurses (88.2% vs. 74.6%, *p* = 0.003) and showed distinct distribution patterns across ICU departments (*p* < 0.001), with neurological ICU having notably higher burnout prevalence. Significant differences were observed in personal characteristics, with the burnout group showing higher rates of marriage (61.3% vs. 35.1%), having children (54.9% vs. 29.8%), and living with partners (53.4% vs. 33.3%) (all *p* < 0.001). Professional factors associated with burnout included higher monthly income (*p* = 0.001), different commuting modes (*p* = 0.009), higher professional titles (*p* < 0.001), and more teaching responsibilities (55.9% vs. 36.0%, *p* = 0.001). Work‐related factors revealed that burnout was associated with inadequate rest time (*p* < 0.001), longer shift patterns (*p* = 0.049), more consecutive working days (median 5.0 vs. 4.0, *p* < 0.001), and higher patient loads (median 3.0 vs. 2.0 patients per shift, *p* < 0.001). Psychometric assessments showed significantly higher stress scores (NSS median 51.5 vs. 32.5, *p* < 0.001), poorer sleep quality (PSQI median 10.0 vs. 8.0, *p* = 0.004), lower resilience (CD‐RISC median 50.0 vs. 80.0, *p* < 0.001), and job satisfaction (MMSS median 90.0 vs. 120.5, *p* < 0.001).

### 3.2. Model Performance Comparison

**FIGURE 2:**
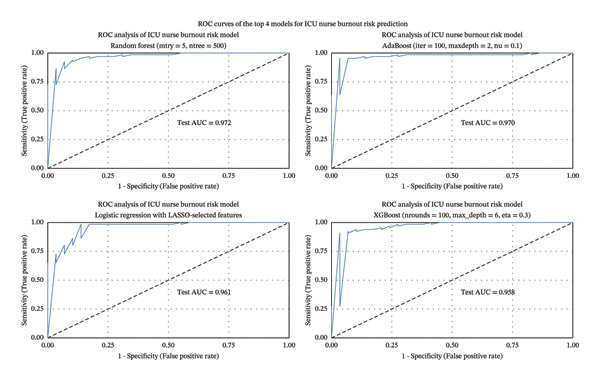
ROC curves of four machine learning models for ICU nurse burnout prediction. The plots display performance of random forest (AUC = 0.972), AdaBoost (AUC = 0.970), logistic regression with LASSO‐selected features (AUC = 0.961), and XGBoost (AUC = 0.958) on the test dataset.

Among the 10 ML algorithms evaluated, the random forest model showed the highest predictive performance for ICU nurse burnout with an AUC of 0.972 (95% CI: 0.944–1.000) and demonstrated consistently strong performance across all other metrics. This was followed by the AdaBoost model (AUC = 0.970, 95% CI: 0.935–1.000), logistic regression (AUC = 0.961, 95% CI: 0.924–0.998), and XGBoost (AUC = 0.958, 95% CI: 0.907–1.000). The ROC curves for these top four performing models are presented in Figure 2. The ROC curves for the remaining six algorithms are available in Supporting Figure 2. Table 2 presents comprehensive performance metrics for all 10 ML models, including AUC (95% CI), recall (95% CI), accuracy (95% CI), F1 score (95% CI), precision (95% CI), and NPV (95% CI), calculated at the optimal cutoff values determined by maximizing the Youden index (sensitivity + specificity − 1).

### 3.3. Determination of the Final Model

**FIGURE 3:**
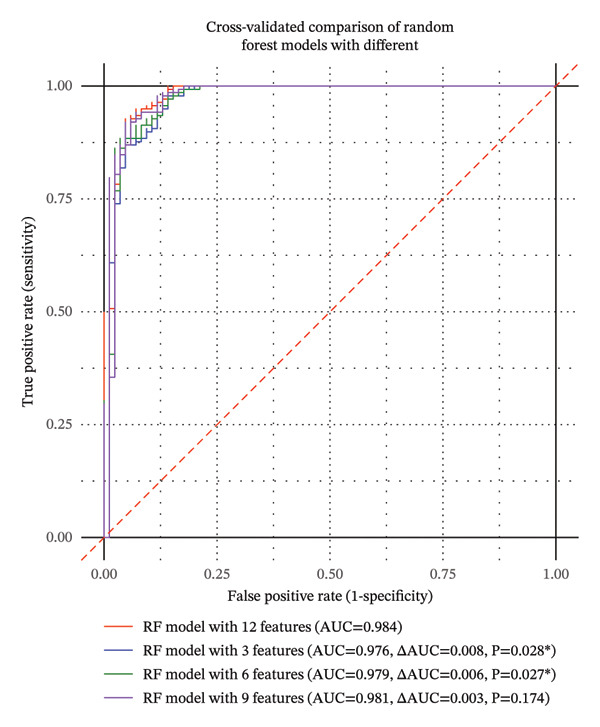
Cross‐validated comparison of random forest models with different feature subsets. ROC curves are based on pooled out‐of‐fold predictions from 10‐fold cross‐validation within the training set. AUC, ΔAUC (versus the 12‐feature model), and DeLong test *p* values are shown. ^∗^ *p* < 0.05 versus the 12‐feature model.

**FIGURE 4:**
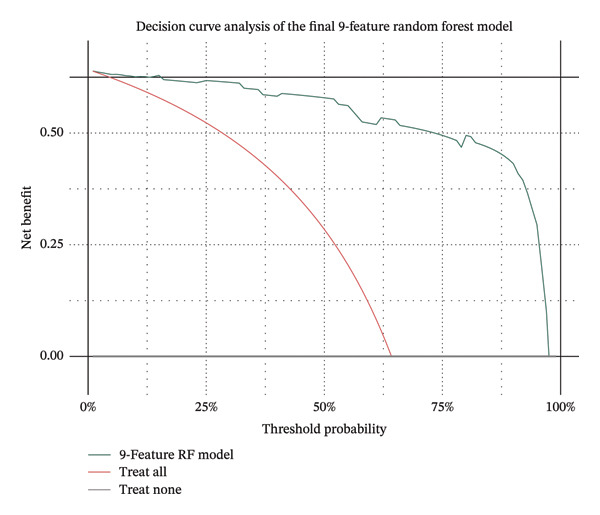
Decision curve analysis of the final 9‐feature random forest model on the test set. The plot shows the net benefit of the model across threshold probabilities, compared with the “treat all” and “treat none” default strategies. The model provided greater net benefit than both reference strategies across the clinically relevant range of threshold probabilities.

In the feature reduction process of the random forest model, we determined that the 9‐feature model provided a suitable balance between model simplicity and predictive capability for ICU nurse burnout. As shown in Figure 3, the 9‐feature RF model achieved a cross‐validated AUC of 0.981 (ΔAUC = 0.003, *p* = 0.174 compared to the 12‐feature model), indicating that removing three features did not significantly affect model performance. The statistical comparison revealed that while there was minimal difference between the 9‐feature and 12‐feature models, reducing to 6 features resulted in a statistically significant decrease in performance (ΔAUC = 0.006, *p* = 0.027). This suggests that the 9‐feature model represents an appropriate compromise that maintains predictive ability while reducing model complexity. The decision curve analysis (Figure 4) further supports this selection. Across most threshold probabilities, and particularly in the clinically relevant ranges, the 9‐feature model showed greater net benefit than the “treat all” and “treat none” reference strategies. Notably, the 9‐feature model maintained reasonable net benefit at higher threshold probabilities, making it potentially useful in various clinical decision contexts. The final 9‐feature random forest model showed these performance metrics on the test set: AUC 0.983. These values suggest that the model may be helpful for identifying nurses with burnout while maintaining a reasonable balance between sensitivity and specificity. Calibration of the final nine‐feature model on the test set was good, with a calibration slope of 0.946, a calibration intercept of 0.115, and a Brier score of 0.054 (Supporting Figure 3), with a slight tendency to underestimate burnout probability in the lower‐to‐middle range.

### 3.4. Model Interpretation

**FIGURE 5:**
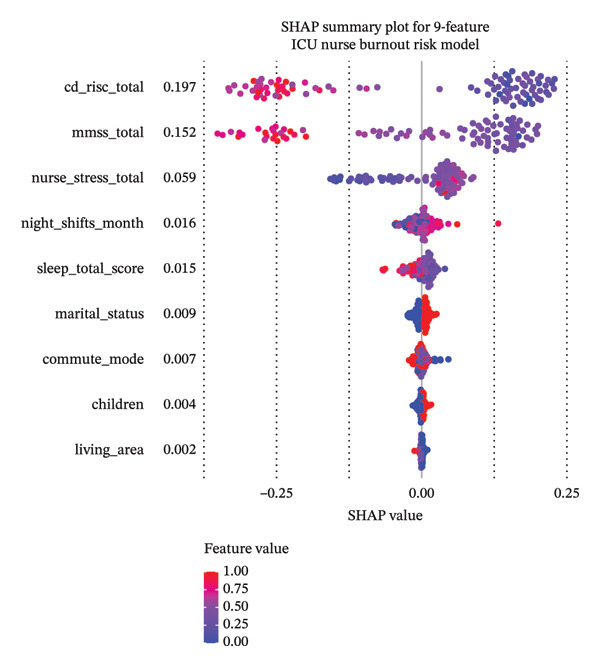
SHAP value analysis showing feature contributions to ICU nurse burnout prediction. Features are ranked by importance (values on left), with dots representing individual observations. Horizontal position indicates positive (right) or negative (left) impact on burnout prediction, while color shows feature value (red = high, blue = low).

The SHAP method was employed to interpret the final random forest model by calculating each variable’s contribution to the prediction outcomes. This approach facilitated both feature‐level global interpretation and individual‐level local explanation of the ICU nurse burnout prediction model. The global interpretation, as illustrated in Figure 5, revealed the distribution of SHAP values across all observations for each feature. The psychological resilience score (cd_risc_total) demonstrated the highest impact on model predictions, with lower values (blue points) generally associated with positive SHAP values, indicating increased burnout risk. Conversely, higher resilience scores (red points) were associated with negative SHAP values, suggesting a protective effect against burnout. Job satisfaction (mmss_total) followed as the second most influential predictor, with lower satisfaction scores contributing positively to burnout prediction.

**FIGURE 6:**
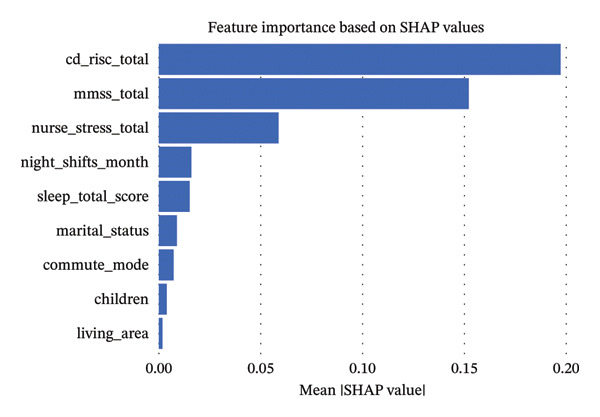
Feature importance ranking for ICU nurse burnout prediction based on mean SHAP values. The bar chart ranks predictors from most important (cd_risc_total) to least important (living_area), quantifying each feature’s average impact on model predictions.

**FIGURE 7:**
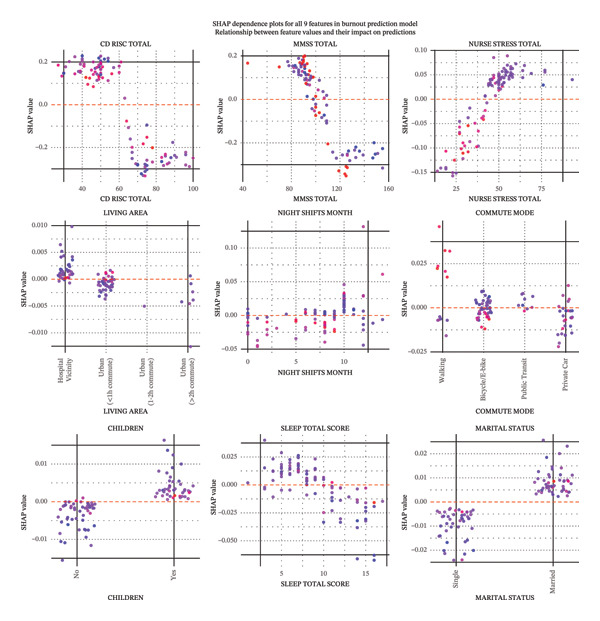
SHAP dependence plots showing relationships between feature values and their impact on ICU nurse burnout predictions. Each subplot displays how individual feature values (*x*‐axis) affect model predictions through SHAP values (*y*‐axis), revealing linear, nonlinear, and categorical feature relationships.

**FIGURE 8:**
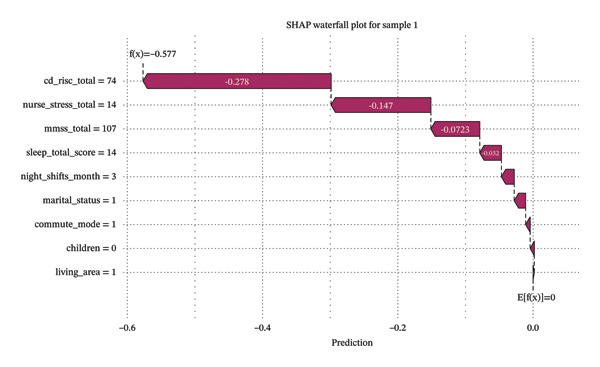
SHAP waterfall plot showing feature contributions for a representative ICU nurse (sample 1). The chart illustrates how each feature value moves the prediction from the base value (*E*[*f*(*x*)] = 0) toward the final predicted value (*f*(*x*) = −0.577), with psychological resilience (cd_risc_total) and nurse stress (nurse_stress_total) having the largest negative contributions.

**FIGURE 9:**
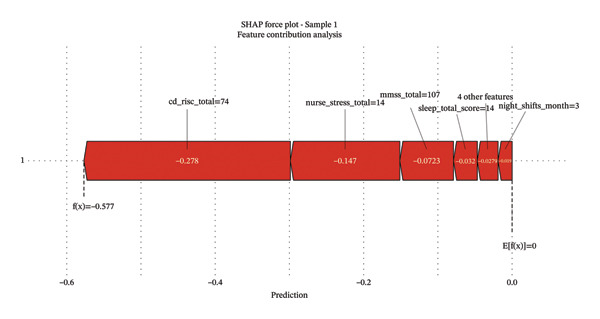
SHAP force plot showing feature contributions for a representative ICU nurse (sample 1). The plot displays how each feature pushes the prediction from the base value (*E*[*f*(*x*)] = 0) toward the final predicted value (*f*(*x*) = −0.577), with psychological resilience (cd_risc_total) and nurse stress (nurse_stress_total) contributing the largest negative effects.

Figure 6 quantified the relative importance of each feature using mean absolute SHAP values. Psychological resilience (cd_risc_total, 0.197) and job satisfaction (mmss_total, 0.152) substantially exceeded other features in importance. Nurse stress level (nurse_stress_total, 0.059) ranked third, followed by monthly night shifts (night_shifts_month, 0.016) and sleep quality (sleep_total_score, 0.015). The remaining four features—marital status, commute mode, number of children, and living area—contributed more modestly to the model, with mean absolute SHAP values below 0.01. The SHAP dependence plots in Figure 7 elucidated the relationship between feature values and their corresponding impact on model predictions for all nine features. The cd_risc_total plot demonstrated a threshold effect, with values below approximately 60 points consistently contributing to increased burnout risk, while values above 70 points had a protective effect. The mmss_total exhibited a negative correlation with burnout risk, though the relationship appeared nonlinear with the most pronounced effects observed at moderate scores. The nurse_stress_total showed a positive association with burnout risk, with higher stress scores consistently increasing predicted burnout probability. The remaining features displayed more complex relationships with prediction outcomes. For instance, night shifts demonstrated variable impacts, while living area categories showed distinct patterns of influence on the model predictions. For individual‐level interpretation, Figure 8 presented a waterfall plot illustrating how each feature contributed to the prediction for a specific nurse. Beginning with a base value of f(x) = ‐0.577, the model incorporated the contributions of each feature to arrive at the final prediction value of E[f(x)] = 0. For this individual, the most substantial contributions came from psychological resilience (cd_risc_total = 74, contribution = −0.278), low stress level (nurse_stress_total = 14, contribution = −0.147), and job satisfaction (mmss_total = 107, contribution = −0.0723). These factors collectively suggested a lower burnout risk for this particular nurse, with smaller contributions from the remaining features. This comprehensive SHAP analysis provided interpretable insights into both the general patterns of feature influence across the population and the specific mechanisms driving individual predictions, potentially informing targeted interventions for ICU nurse burnout prevention (see Figure 9).

## 4. Discussion

This study developed a ML model for predicting burnout among ICU nurses, with attention to unbiased evaluation, principled feature selection, and interpretability. Strict separation of training and test data prevented information leakage during feature selection and hyperparameter tuning, an issue that has been highlighted in healthcare prediction research [52]. Combining LASSO regularization with sequential SHAP‐guided reduction produced a parsimonious nine‐predictor random forest model that retained the discrimination of the full twelve‐predictor model while being simpler to apply [53]. SHAP analysis identified psychological resilience, job satisfaction, and nurse stress as the most influential predictors, pointing to plausible targets for intervention. Decision curve analysis indicated that the nine‐predictor model offered net benefit over “treat all” and “treat none” strategies across clinically relevant threshold probabilities, although its real‐world utility will need to be confirmed in independent samples.

Several patterns emerged from the SHAP dependence plots that merit comment. The three top predictors—psychological resilience, job satisfaction, and nurse stress—each showed evidence of nonlinear, threshold‐like relationships with burnout risk rather than smooth linear effects. For resilience and job satisfaction, lower scores were associated with markedly higher predicted burnout, while above approximately the upper‐quartile range the protective effect attenuated. Nurse stress showed the inverse pattern, with predicted risk rising sharply once moderate stress was exceeded [54, 55]. The remaining six predictors—night shift frequency, commuting time and mode, marital status, parenthood, sleep quality, and residential proximity—contributed more modestly and with greater individual variability [56, 57], consistent with their secondary ranking in the global feature importance. Together, these patterns suggest that burnout risk in this ICU nurse sample reflects an interplay of psychological, work‐related, and lifestyle factors rather than any single dominant cause. The specific cutpoints suggested by the dependence plots should be regarded as data‐driven and exploratory; their stability and clinical applicability would need to be examined in independent samples before being used to inform individual screening decisions.

Direct comparison of our discrimination metrics with prior burnout‐prediction studies is difficult, as previous models have used different outcome definitions, predictor sets, and validation strategies. Reported AUCs in this literature have varied substantially: A recent model predicting primary care physician burnout from electronic health record use achieved an AUC of around 0.74 [58], and earlier nurse‐focused models using logistic regression have reported lower discrimination [59]. The numerically higher AUC observed in our study likely reflects, at least in part, the use of an ensemble algorithm together with a tightly defined ICU nurse sample, but it may also be partly explained by the optimism inherent in internal test‐set evaluation. External validation in independent cohorts will be needed before drawing conclusions about relative model performance.

The prominence of psychological resilience and job satisfaction as protective factors is broadly consistent with the existing literature. Resilience has been linked to lower burnout in critical care and emergency nursing populations [60], and longitudinal evidence indicates that job resources such as supervisor support reduce EE over time [61]. The high ranking of nurse stress in our model is also in line with a Chinese ICU nurse study that reported a strong stress–burnout association [62]. The dependence‐plot cutpoints (resilience around 60 and job satisfaction around 80) suggest where the protective effects begin to attenuate in this sample but should be regarded as exploratory and would need confirmation in independent ICU cohorts before being used to guide screening.

Some of our findings differ from those reported in other settings and may reflect contextual factors specific to Chinese tertiary hospitals. In our model, being married and having children were associated with higher rather than lower burnout risk, in contrast to a systematic review of emergency‐nurse burnout that found no consistent relationship between marital status and burnout [63]. A plausible explanation lies in work–family conflict in the Chinese ICU context: Long shifts, limited family leave, and traditional caregiving expectations placed primarily on women may turn family responsibilities into an additional source of strain rather than a buffer. A previous Chinese study similarly reported higher burnout among nurses with children [64]. These observations suggest that risk profiles for ICU nurse burnout are likely shaped by local working conditions and cultural expectations and reinforce the need to retest the model in other Chinese settings and in non‐Chinese ICU samples.

Although prospective and external evidence is needed before the model can be used in practice, the consistent ranking of psychological resilience, job satisfaction, and nurse stress across our analyses points to plausible focuses for future intervention work. Programs that strengthen resilience, improve perceived job satisfaction (for example, through supervisor support and more equitable workload distribution), and reduce occupational stress through staffing and shift‐scheduling changes have all been studied in nursing populations and would be reasonable candidates to evaluate in ICU samples. The cutpoints suggested by the dependence plots are not yet ready to guide individual screening decisions but may help generate hypotheses about where intervention thresholds could be tested. Because our design is cross‐sectional, we cannot determine whether these factors are causes, consequences, or both; intervention studies and longitudinal cohorts will be needed to establish whether modifying them changes burnout incidence. Practical deployment of any such model would also depend on integration with existing workflows, staff training, and clear referral pathways and on recognizing that burnout is sustained by organizational conditions as much as by individual characteristics.

Implications for nurse managers. Because the most influential predictors in our model—psychological resilience, job satisfaction, nurse stress, night‐shift frequency, and sleep—are shaped substantially by working conditions, the findings point primarily to organizational rather than individual levers. Nurse managers could use models of this kind to identify units or groups at higher risk and to prioritize structural responses, rather than placing the onus on individual nurses to “cope better.” At the organizational level, this includes ensuring adequate staffing and manageable nurse‐to‐patient ratios to reduce chronic stress [65]; designing schedules that protect intershift recovery, for example, by limiting consecutive night shifts and guaranteeing sufficient rest between shifts; and strengthening job satisfaction through supervisor support, recognition, equitable workload distribution, and opportunities for professional development. Resilience is best supported not by exhorting nurses to be more resilient but by providing organizational resources—mentorship, accessible psychological support, and a supportive team climate—that make resilience sustainable. Framed in this way, the model is a tool to guide managerial and organizational burnout‐prevention efforts, consistent with evidence that burnout is driven by the work environment at least as much as by individual characteristics [66].

### 4.1. Limitations

This study has several important limitations that warrant consideration. First, the cross‐sectional design fundamentally limits causal inference capabilities, offering only a snapshot of associations between predictors and burnout at a single time point without establishing temporal precedence necessary for causality. Sampling limitations must also be acknowledged, as recruitment from multiple tertiary hospitals in China may restrict generalizability to other healthcare systems with different organizational structures, cultural contexts, and resource allocations. The exclusion criteria, while methodologically necessary, may have introduced selection bias by systematically removing certain nurse subpopulations. A further consideration concerns the operationalization of burnout. Although we required simultaneous elevation across all three MBI dimensions—a stringent criterion consistent with Maslach’s view of burnout as a multidimensional syndrome—reducing the construct to a binary outcome inevitably discards information about severity and dimensional profile, and nurses scoring near the cutoffs may be misclassified. Modeling burnout as a continuous score or as distinct dimensional profiles in future work could capture this nuance. LASSO regression used for feature selection prioritizes predictive efficiency over theoretical importance, potentially excluding variables with substantive relevance but weaker statistical signals. Despite implementing cross‐validation and maintaining strict data isolation protocols, the relatively moderate sample size (*N* = 318) may limit the stability of complex ML models when applied to new populations. External validation in independent samples and across different ICU settings is therefore essential before the model can inform clinical practice. The inverse frequency weighting method used to address class imbalance, while methodologically sound, represents just one of several possible correction strategies that might have different impacts on model performance. Finally, the study’s focus on individual‐level predictors may inadvertently frame burnout as primarily an individual phenomenon rather than acknowledging systemic contributors within healthcare organizations. This limitation is particularly relevant when considering potential intervention strategies, which may require multilevel approaches targeting both individual resilience factors and structural workplace conditions. Future research would benefit from prospective, longitudinal designs incorporating both individual and organizational measures, mixed‐methods approaches to capture qualitative dimensions of burnout experience, and intervention studies evaluating whether addressing identified predictors effectively reduces burnout incidence in ICU nursing populations. Calibration was assessed in addition to discrimination and was generally good, with a minor under‐estimation of risk at lower predicted probabilities; because inverse‐frequency weighting and the modest test‐set size can affect probability calibration, recalibration in larger, external cohorts is a useful direction for future work.

A further limitation concerns the assessment of recovery. Although our predictor set captured several aspects of the work–recovery cycle—monthly night‐shift frequency, adequacy of intershift rest, number of consecutive working days, shift length, and sleep quality—we did not administer a dedicated, validated measure of intershift recovery such as the Occupational Fatigue Exhaustion/Recovery (OFER) scale [67]. Contemporary occupational‐stress models emphasize that the failure to recover between consecutive shifts, rather than fatigue or stress per se, may be an important driver of long‐term health consequences. Because the recovery construct was not measured directly, it cannot be reconstructed from the present data; future prospective studies should incorporate the OFER scale (a validated Chinese version is available) [68] and examine whether intershift recovery mediates the relationship between shift‐related demands and burnout.

In addition, although the MBI remains the most widely used and validated burnout instrument, it is a psychological self‐report measure and does not capture the neurophysiological dimension of chronic stress described by allostatic‐load frameworks; the validity of its reduced‐personal‐accomplishment subscale, in particular, continues to be debated. We mitigated overreliance on any single dimension by defining burnout conjunctively across all three MBI dimensions, but future work modeling burnout dimensionally—for example, focusing on EE, the most temporally stable subscale—or integrating physiological indicators would offer a more complete picture.

## 5. Conclusion

This study developed an interpretable ML model—combining LASSO feature selection with a random forest classifier—to identify current burnout among ICU nurses. The model showed reasonable performance on the test set, and SHAP analysis identified psychological resilience, job satisfaction, and nurse stress as the most influential predictors, each showing threshold‐like effects, with smaller contributions from factors such as night shifts and sleep quality. By providing both population‐ and individual‐level interpretability, the model may help generate hypotheses about where intervention thresholds could be tested. However, the cross‐sectional design and reliance on self‐report limit causal inference, and prospective and external validation are needed before clinical use. For nurse managers, the findings suggest that burnout prevention is best pursued through organizational measures—adequate staffing, recovery‐protective scheduling, and support for job satisfaction and resilience—rather than by placing responsibility on individual nurses.

## Funding

No funding was received for this manuscript.

## Conflicts of Interest

The authors declare no conflicts of interest.

## Supporting Information

Additional supporting information can be found online in the Supporting Information section.

## Supporting information


**Supporting Information** This study includes the following supplementary materials that provide additional support for the main research findings: supplementary figure descriptions. This document provides descriptions for the supplementary figures related to the ICU nurse burnout risk prediction model study. Supplementary figures overview the following figures and provide additional analytical insights that support the main findings presented in our research. Both figures are compiled into a single document for easier review. Supporting Figure 1. SHAP summary plot ranking all features in the ICU nurse burnout risk model. Features are ordered by importance (values on left), with dots representing individual observations. The plot shows cd_risc_total (resilience), mmss_total (likely satisfaction), and nurse_stress_total as the top predictors. Red dots (high values) of resilience and satisfaction scores push predictions toward lower burnout risk (negative SHAP values). Supporting Figure 2. Comparative ROC analysis of six different models for ICU nurse burnout risk assessment. Each panel shows the ROC curve (true positive rate vs. false positive rate) for a different model configuration, with AUC values ranging from 0.933 to 0.950. Supporting Figure 3. Calibration curve for the final nine‐feature random forest model on the held‐out test set. The solid line shows the loess‐smoothed relationship between predicted probability and observed proportion of burnout; the shaded band is the 95% CI, and the dashed line denotes perfect calibration. The rug shows the distribution of predicted probabilities. Calibration slope = 0.946, intercept = 0.115, and Brier score = 0.054. Supporting File 1. Hyperparameter settings and grid search ranges for machine learning models: Supporting File 1 presents a comprehensive documentation of the hyperparameter optimization process for the 10 machine learning algorithms employed in predicting burnout syndrome among intensive care unit nurses. This document delineates the systematic grid search methodology utilized to identify optimal parameter configurations for each predictive model. For each algorithm, we specify the full range of hyperparameters subjected to tuning, their respective search spaces, and the final optimized values derived through k‐fold cross‐validation procedures. The file contains detailed information regarding both modified parameters and those maintained at their default settings, accompanied by the theoretical rationale underlying specific parameter choices where applicable.

## Data Availability

The data that support the findings of this study are available on request from the corresponding author. The data are not publicly available due to privacy or ethical restrictions.
