# Supplementary material for: Development and Internal Validation of Interpretable Machine Learning Models for Identifying Burnout Syndrome Among Intensive Care Unit Nurses
Source: J Nurs Manag. 2026 Jul 19;2026:6835251. doi: 10.1155/jonm/6835251 (PMC13382357; doi:10.1155/jonm/6835251)
Supplement: Supplementary file 1 — Supporting Information This study includes the following supplementary materials that provide additional support for the main research findings: supplementary figure descriptions. This document provides descriptions for the supplementary figures related to the ICU nurse burnout risk prediction model study. Supplementary figures overview the following figures and provide additional analytical insights that support the main findings presented in our research. Both figures are compiled into a single document for easier review. Supporting Figure 1. SHAP summary plot ranking all features in the ICU nurse burnout risk model. Features are ordered by importance (values on left), with dots representing individual observations. The plot shows cd_risc_total (resilience), mmss_total (likely satisfaction), and nurse_stress_total as the top predictors. Red dots (high values) of resilience and satisfaction scores push predictions toward lower burnout risk (negative SHAP values). Supporting Figure 2. Comparative ROC analysis of six different models for ICU nurse burnout risk assessment. Each panel shows the ROC curve (true positive rate vs. false positive rate) for a different model configuration, with AUC values ranging from 0.933 to 0.950. Supporting Figure 3. Calibration curve for the final nine‐feature random forest model on the held‐out test set. The solid line shows the loess‐smoothed relationship between predicted probability and observed proportion of burnout; the shaded band is the 95% CI, and the dashed line denotes perfect calibration. The rug shows the distribution of predicted probabilities. Calibration slope = 0.946, intercept = 0.115, and Brier score = 0.054. Supporting File 1. Hyperparameter settings and grid search ranges for machine learning models: Supporting File 1 presents a comprehensive documentation of the hyperparameter optimization process for the 10 machine learning algorithms employed in predicting burnout syndrome among intensive care unit nurses. This [file JONM-2026-6835251-s001.zip › supplementary figure descriptions.docx]

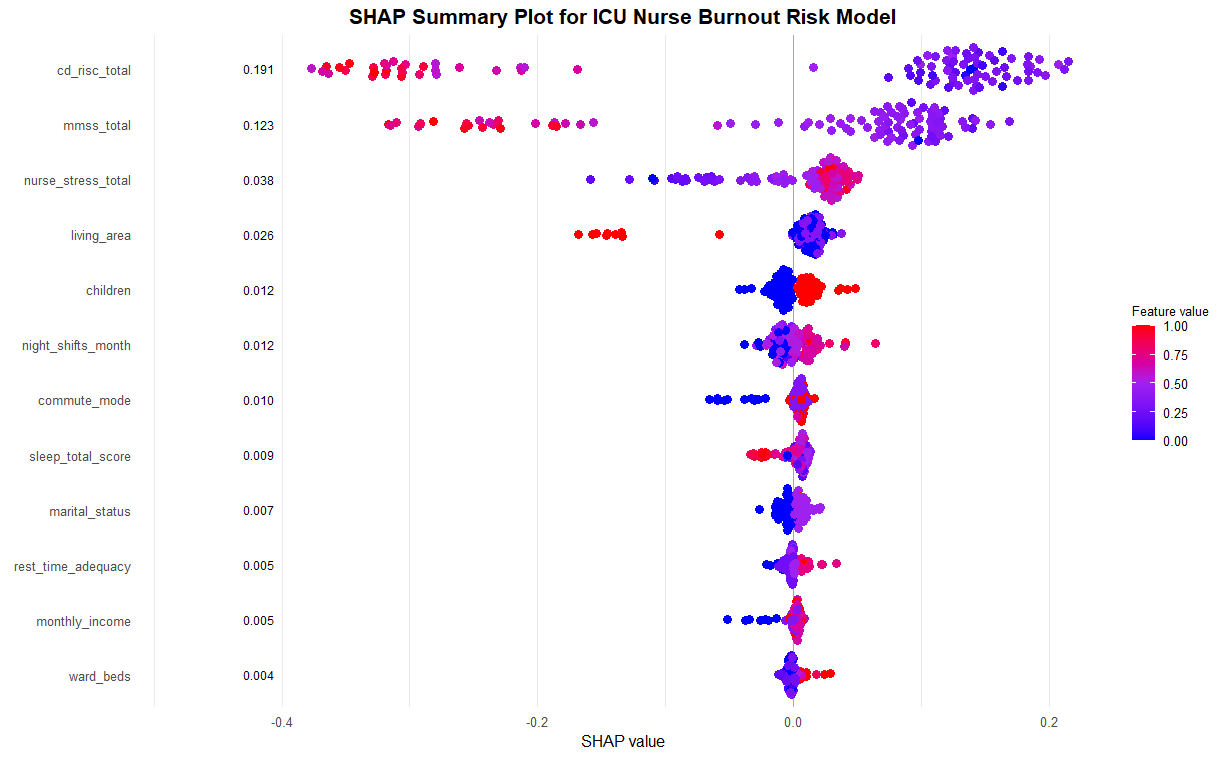


Supplementary Figure 1. SHAP summary plot ranking all features in the ICU nurse burnout risk model. Features are ordered by importance (values on left), with dots representing individual observations. The plot shows cd_risc_total (resilience), mmss_total (likely satisfaction), and nurse_stress_total as the top predictors. Red dots (high values) of resilience and satisfaction scores push predictions toward lower burnout risk (negative SHAP values).


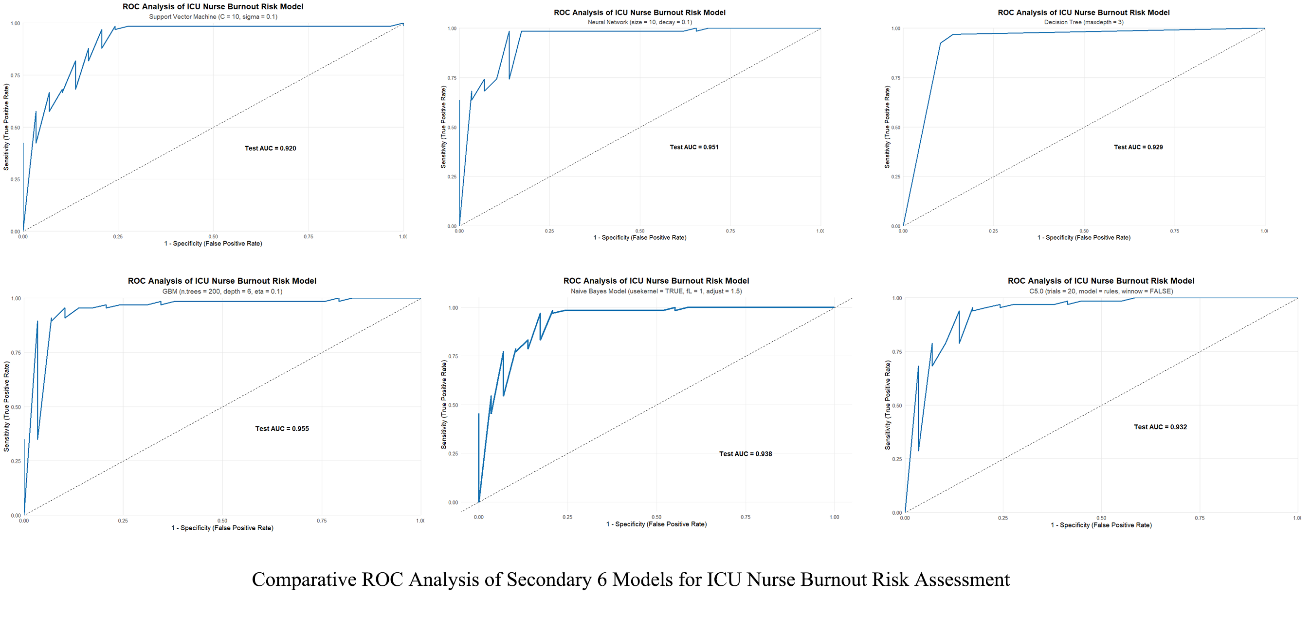


Supplementary Figure 2. Comparative ROC analysis of six different models for ICU nurse burnout risk assessment. Each panel shows the ROC curve (True Positive Rate vs. False Positive Rate) for a different model configuration, with AUC values ranging from 0.933 to 0.950.


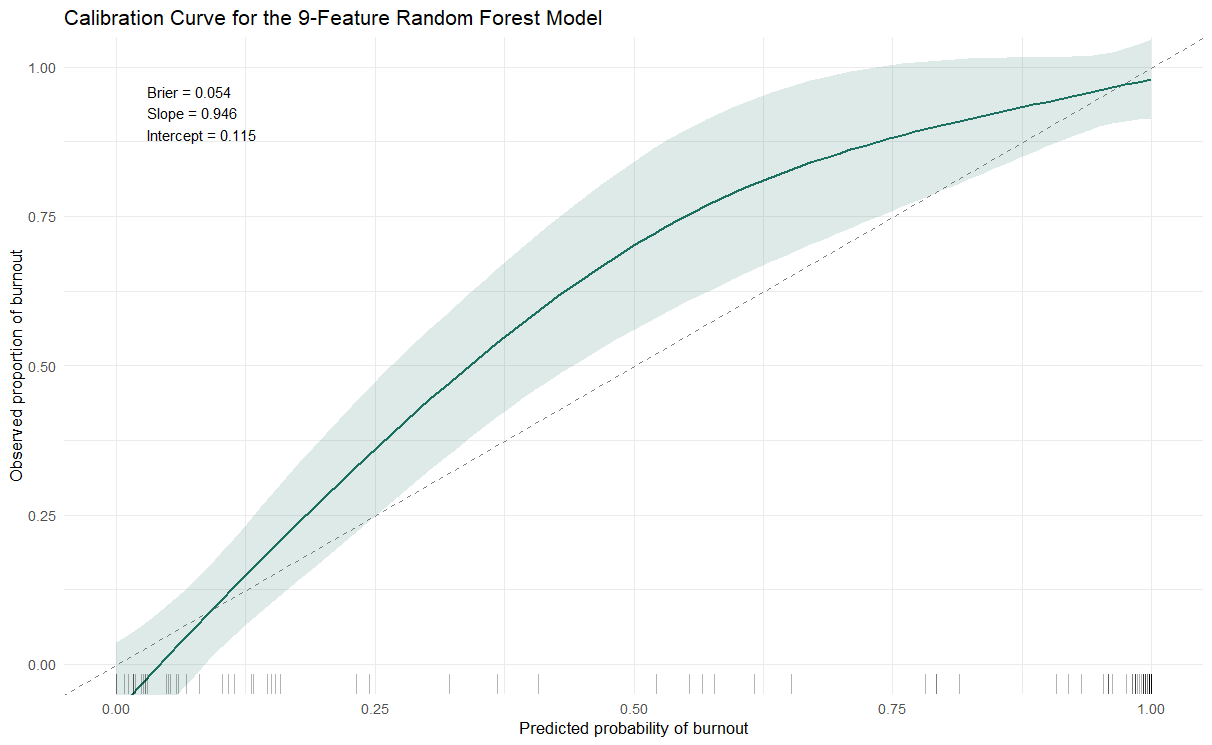


Supplementary Figure 3. Calibration curve for the final nine-feature random forest model on the held-out test set. The solid line shows the loess-smoothed relationship between predicted probability and observed proportion of burnout; the shaded band is the 95% confidence interval and the dashed line denotes perfect calibration. The rug shows the distribution of predicted probabilities. Calibration slope = 0.946, intercept = 0.115, Brier score = 0.054.
