# Supplementary material for: Development and Internal Validation of Interpretable Machine Learning Models for Identifying Burnout Syndrome Among Intensive Care Unit Nurses
Source: J Nurs Manag. 2026 Jul 19;2026:6835251. doi: 10.1155/jonm/6835251 (PMC13382357; doi:10.1155/jonm/6835251)
Supplement: Supplementary file 1 — Supporting Information This study includes the following supplementary materials that provide additional support for the main research findings: supplementary figure descriptions. This document provides descriptions for the supplementary figures related to the ICU nurse burnout risk prediction model study. Supplementary figures overview the following figures and provide additional analytical insights that support the main findings presented in our research. Both figures are compiled into a single document for easier review. Supporting Figure 1. SHAP summary plot ranking all features in the ICU nurse burnout risk model. Features are ordered by importance (values on left), with dots representing individual observations. The plot shows cd_risc_total (resilience), mmss_total (likely satisfaction), and nurse_stress_total as the top predictors. Red dots (high values) of resilience and satisfaction scores push predictions toward lower burnout risk (negative SHAP values). Supporting Figure 2. Comparative ROC analysis of six different models for ICU nurse burnout risk assessment. Each panel shows the ROC curve (true positive rate vs. false positive rate) for a different model configuration, with AUC values ranging from 0.933 to 0.950. Supporting Figure 3. Calibration curve for the final nine‐feature random forest model on the held‐out test set. The solid line shows the loess‐smoothed relationship between predicted probability and observed proportion of burnout; the shaded band is the 95% CI, and the dashed line denotes perfect calibration. The rug shows the distribution of predicted probabilities. Calibration slope = 0.946, intercept = 0.115, and Brier score = 0.054. Supporting File 1. Hyperparameter settings and grid search ranges for machine learning models: Supporting File 1 presents a comprehensive documentation of the hyperparameter optimization process for the 10 machine learning algorithms employed in predicting burnout syndrome among intensive care unit nurses. This [file JONM-2026-6835251-s001.zip › Supplementary File 1.docx]

**Hyperparameter Settings and Grid Search Ranges for Machine Learning Models**

**Random Forest Hyperparameter Grid Search Results**

**Search Parameters**

mtry (number of variables randomly sampled at each split)

Search range: {1, 2, 3, 4, 5, 6}

Note: With 12 predictors total, this covers 8% to 50% of available features

**Optimal Values**

mtry = 5(final selected value)

Other parameters kept at default values:

min.node.size = 1 (default)

splitrule = "gini" (default)

**SVM Hyperparameter Grid Search Results**

**Search Parameters**

C (cost parameter)

Search range: {0.1, 1.0, 10.0, 100.0}

Controls the trade-off between decision boundary smoothness and misclassification penalty

sigma (RBF kernel parameter)

Search range: {0.01, 0.1, 1.0}

Determines the influence radius of support vectors in the feature space

**Optimal Values**

C = 10(final selected value)

sigma = 0.1(final selected value)

**XGBoost Hyperparameter Grid Search Results**

**Search Parameters**

eta (learning rate)

Search range: {0.1, 0.3}

Controls step size shrinkage used to prevent overfitting

max_depth (maximum tree depth)

Search range: {3, 6}

Controls complexity of individual trees

**Fixed Parameters**

nrounds = 100 (number of boosting rounds)

gamma = 0 (minimum loss reduction for partition)

colsample_bytree = 0.75 (fraction of features per tree)

min_child_weight = 1 (minimum sum of instance weight in child)

subsample = 0.75 (fraction of training instances used)

**Optimal Values**

eta = 0.3 (final selected value)

max_depth = 6 (final selected value)

**GBM Hyperparameter Grid Search Results**

**Search Parameters**

shrinkage (learning rate)

Search range: {0.05, 0.10}

Controls contribution of each tree to the final model

interaction.depth (tree depth)

Search range: {4, 6}

Controls complexity of individual trees

n.trees (number of boosting iterations)

Search range: {100, 200}

Total number of trees in the ensemble

**Fixed Parameters**

n.minobsinnode = 10 (minimum number of observations in terminal nodes)

**Optimal Values**

n.trees = 100 (final selected value)

interaction.depth = 4 (final selected value)

shrinkage = 0.1 (final selected value)

n.minobsinnode = 10 (fixed value)

**Neural Network Hyperparameter Grid Search Results**

**Search Parameters**

**size (hidden layer neurons)**

Search range: {5, 10}

Controls model complexity and representational capacity

**decay (weight regularization)**

Search range: {0.01, 0.10}

Controls the strength of L2 regularization to prevent overfitting

**Fixed Parameters**

**maxit** = 500 (maximum number of iterations during training)

**Optimal Values**

**size = 10** (final selected value)

**decay = 0.1** (final selected value)

**AdaBoost Hyperparameter Grid Search Results**

**Search Parameters**

**iter (number of weak learners)**

Search range: {50, 100}

Controls the number of decision trees in the ensemble

**maxdepth (decision tree depth)**

Search range: {1, 2}

Controls complexity of individual trees

**nu (learning rate)**

Fixed value: 0.1

Controls contribution of each tree to the final prediction

**Optimal Values**

**iter = 100** (final selected value)

**maxdepth = 2** (final selected value)

**nu = 0.1** (fixed value)

**Decision Tree Hyperparameter Grid Search Results**

**Search Parameters**

**maxdepth (maximum tree depth)**

Search range: {3, 5, 8, 10, 15}

Controls the maximum allowed depth of decision trees

**Optimal Values**

**maxdepth = 3** (final selected value)

**Naive Bayes Hyperparameter Grid Search Results**

**Search Parameters**

**usekernel (kernel density estimation)**

Search range: {TRUE, FALSE}

Controls whether to use kernel density estimation for continuous variables

**fL (Laplace smoothing)**

Search range: {0.0, 0.5, 1.0, 2.0}

Controls smoothing for zero probability problems

**adjust (bandwidth adjustment)**

Search range: {0.5, 1.0, 1.5, 2.0}

Controls smoothness of kernel density estimation

**Optimal Values**

**usekernel = TRUE** (final selected value)

**fL = 1** (final selected value)

**adjust = 1.5** (final selected value)

**C5.0 Hyperparameter Grid Search Results**

**Search Parameters**

**trials (boosting iterations)**

Search range: {1, 5, 10, 20, 30, 50}

Controls number of boosting iterations for ensemble construction

**model (algorithm type)**

Options: {"tree", "rules"}

Determines whether to use tree-based or rule-based classification

**winnow (feature selection)**

Options: {TRUE, FALSE}

Controls whether to use feature selection during training

**Optimal Values**

**trials = 20** (final selected value)

**model = rules** (final selected value)

**winnow = FALSE** (final selected value)

Supplementary File 1. Hyperparameter Settings and Grid Search Ranges for Machine Learning Models: Supplementary File 1 presents a comprehensive documentation of the hyperparameter optimization process for the ten machine learning algorithms employed in predicting burnout syndrome among intensive care unit nurses. This document delineates the systematic grid search methodology utilized to identify optimal parameter configurations for each predictive model. For each algorithm, we specify the full range of hyperparameters subjected to tuning, their respective search spaces, and the final optimized values derived through k-fold cross-validation procedures. The file contains detailed information regarding both modified parameters and those maintained at their default settings, accompanied by the theoretical rationale underlying specific parameter choices where applicable.
